# A highly recurrent RPS27 5'UTR mutation in melanoma

**DOI:** 10.18632/oncotarget.2048

**Published:** 2014-06-01

**Authors:** Ken Dutton-Regester, Jared J. Gartner, Rafi Emmanuel, Nouar Qutob, Michael A. Davies, Jeffrey E. Gershenwald, William Robinson, Steven Robinson, Steven A. Rosenberg, Richard A. Scolyer, Graham J. Mann, John F. Thompson, Nicholas K. Hayward, Yardena Samuels

**Affiliations:** ^1^ QIMR Berghofer Medical Research Institute, Brisbane, QLD, Australia; ^2^ National Cancer Institute, NIH, MD, USA; ^3^ Department of Molecular Cell Biology, Weizmann Institute of Science, Rehovot, Israel; ^4^ The University of Texas MD Anderson Cancer Center, Houston, TX, USA; ^5^ University of Colorado School of Medicine, Aurora, CO, USA; ^6^ Melanoma Institute of Australia (formerly the Sydney Melanoma Unit), North Sydney,NSW, Australia; ^7^ Tissue Pathology and Diagnostic Oncology, Royal Prince Alfred Hospital, Camperdown, NSW, Australia; ^8^ Discipline of Pathology, Sydney Medical School, The University of Sydney, NSW, Australia; ^9^ University of Sydney at Westmead Millenium Institute, Westmead, NSW, Australia; ^10^ Departments of Melanoma and Surgical Oncology, and Royal Prince Alfred Hospital, Sydney, New South Wales, Australia; ^11^ Discipline of Surgery, Sydney Medical School, The University of Sydney, Sydney, New South Wales, Australia

**Keywords:** Melanoma, somatic mutation, RPS27, exome sequencing, 5' untranslated region

## Abstract

The incidence of melanoma continues to rise globally and is increasing at a rate greater than any other cancer. To systematically search for new genes involved in melanomagenesis, we collated exome sequencing data from independent melanoma cohort datasets, including those in the public domain. We identified recurrent mutations that may drive melanoma growth, survival or metastasis, and which may hold promise for the design of novel therapies to treat melanoma. These included a frequent recurrent (i.e. hotspot) mutation in the 5' untranslated region of RPS27 in ~10% of samples. We show that the mutation expands the 5'TOP element, a motif known to regulate the expression of most of the ribosomal protein family, to which RPS27 belongs, and thus might sensitize the mutated transcript to growth-mediated regulation. This finding highlights not only the important role of non-protein coding genetic aberrations in cancer development but also their potential as novel therapeutic targets.

## INTRODUCTION

Melanoma is the deadliest form of human skin cancer, accounting for 70% of skin cancer related deaths, with approximately 48,000 fatalities annually worldwide [1,2]. Candidate gene analyses have been powerful in identifying mutations, such as BRAF V600E, which has become a successful target for the FDA-approved inhibitor, vemurafenib. However, approximately 50% of patients do not harbor the mutation and some patients with a BRAF mutation do not respond to the drug, or quickly acquire resistance. As such, there is a strong focus to improve the therapeutic strategies for late-stage melanoma. Current approaches include the investigation of various drug combinations, scheduling and dosage regimens, and the development of new therapeutics through the identification of novel drug targets [6-12]. Recent large scale exome and genome sequencing studies have rapidly expanded the known genetic drivers involved in melanomagenesis [13-25] Despite this, it is likely a number of additional driver genes have yet to be discovered as scaling genetic studies with larger sample sizes will reveal novel genes mutated at clinically important but lower frequencies [26]. Here, we have used a systematic approach to identify additional candidate melanoma genes in an unbiased fashion.

## RESULTS

One way to identify driver mutations is to look for those that are recurrent across multiple samples. Recently, several next generation sequencing studies have been published on melanoma. We have utilized the somatic mutation data from 4 of these studies as well as unpublished data we have generated (Supplementary Figure 1). The merging of these datasets substantially increased the number of samples available to review, thus increasing the chances of identifying low frequency recurrent mutations. As the published studies were all performed at different times and used different procedures to annotate their variants, several steps had to be applied to get the data into the same format. In some cases the data needed to be converted to the more recent genomic build (hg19) and redundant samples needed to be removed. Once a list of mutations was established from each study, the positional data and changes were formatted and annotated using the web-based version of oncotator (http://www.broadinstitute.org/oncotator). Many of these studies assessed a combination of both fresh tissue and cell line samples of various melanoma subtypes, however as the focus of our study was on cutaneous malignant melanoma, the subtypes of acral, mucosal and uveal melanoma were removed from the newly annotated data. We also removed any samples where the initial publication did not include a matched normal sample. The resulting output left 246 metastatic melanoma samples and their annotated somatic mutation data to review (Supplementary Table 1).

We limited the scope of our investigation to 178 mutations occurring at the same chromosomal position in 4 or more of the 246 samples (Supplementary Table 2). As expected, this list included well documented frequent oncogenic drivers of melanoma, including hotspot mutations in *BRAF*, *NRAS*, *RAC1*, *PPP6C*, *TRRAP, MAP3K5* and *BCL2L12 [5, 17, 27-29]*. Of the 178 mutations, 136 are missense, 4 are nonsense, 21 are silent and 17 occur in 5' UTRs or within splicing regions (Supplementary Table 2).

Of the 178 hotspot mutations identified in the collated next-generation sequencing dataset, we prioritized those that most frequently appeared, occurring in 5 or more samples, or which occurred at least 4 times in genes with a previous association with tumorigenesis. We excluded known hotspot mutations in *BRAF*, *NRAS*, *RAC1*, *BCL2L12*, *PPP6C*, *TRRAP*, and *MAP3K5* from further analysis since these have been thoroughly investigated. To validate the mutations and more accurately determine prevalence rates, we screened these new candidate hotspots in a validation panel that consisted of up to 489 melanoma cell lines or tumors (Supplementary Table 3). Of these, 234 had matching normal DNA that enabled us to unambiguously determine somatic mutation status.

A total of 64 mutations were selected for follow up screening using the Sequenom MassARRAY® system (Supplementary Table 4). Three of these failed the design process or could not be validated by Sanger sequencing, most likely as a result of these mutations being false positive calls in the exome data due to repetitive regions of the genome. Eventually, 61 mutations were screened in 489 samples (Supplementary Table 5). Mutation detection was determined using a minimum 10% threshold of the mutant allele peak and were all reviewed manually.

From our validation results, the mutation seen at the highest frequency was *RPS27* (47 of 489, or 9.6%) (Table 1 and Supplementary Table 6). Furthermore, we screened a panel of 34 cell lines of various tumor types (Supplementary Table 7 and Supplementary Table 8); however no mutations were identified, suggesting the mutation might be specific to melanoma.

**Table 1:** *RPS27* gene is mutated at high frequency in melanoma

Mutation	# of Mut samples	% of mutant (n= 489)
RPS27_1_5'UTR	47	9.61
RBM22_3_5'UTR	28	6.22
CHCHD2_87_5'UTR	20	4.44
UMPS_22_5'UTR	20	4.44
OR4M2_S268F	15	3.07
OR4N2_G41E	11	2.25
RQCD1_P131L	10	2.22
RPL37_116_5'UTR	9	2
ISX_R86C	9	1.84

**Figure 1:**
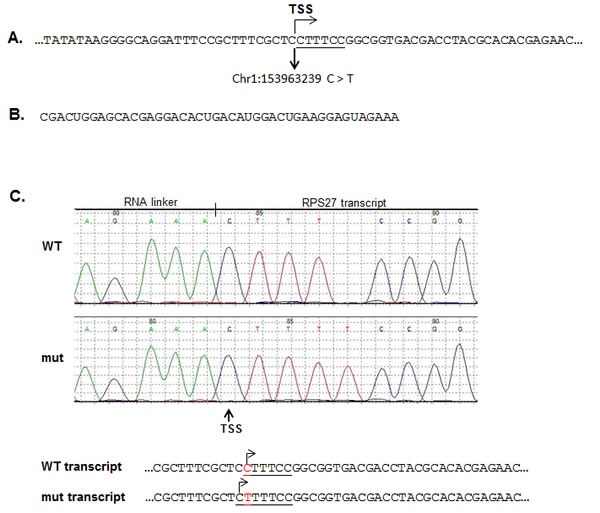
The RPS27 recurrent mutation alters the transcription starting site (TSS) of the RPS27 gene A. Description of the genomic sequence flanking the recurrent mutation of the *RPS27* gene. The 5'TOP element sequence is underlined. To determine the effect of the C > T recurrent mutation on the TSS of the *RPS27* gene, RACE analysis was performed using the RNA linker described in B. cDNA was prepared from the ligated mRNA and amplified using a Forward primer located in the linker and a *RPS27* specific Reverse primer. The amplified products were cloned in pGEM-T easy vector. Colonies were picked and sequenced. A representative chromatography and the genomic sequence of the 5' terminus of the transcripts is described in C. The 5'TOP element is underlined, the TSS is designated with arrows and the C > T substitution is highlighted with red. WT: wild-type; mut: mutant.

The recurrent mutation in the *RPS27* gene is located in the 5'UTR at position Chr1:153963239 and results in a cytidine to thymidine change. According to the Data Base of Transcriptional Start Site (DBTSS, http://dbtss.hgc.jp), this position is the major transcriptional start site of the *RPS27* gene. Comparable to most genes in the family of ribosomal proteins, *RPS27* harbors a 5' terminal oligopyrimidine (5'TOP) tract, which is required for the translational control of the protein in a growth-dependent manner [30]. It has been shown that for effective translational regulation, the 5'TOP element must begin with a cytidine residue at the 5' terminus of the mRNA, followed by an uninterrupted stretch of 4 to 15 pyrimidines [31]. Moreover, a single substitution of cytidine with uridine, at the cap position is sufficient to abolish the translational control of the 5'TOP element [32]. The recurrent mutation in the *RPS27* gene changes the cytidine at the cap site with a thymidine (Figure 1A). In case the transcription start site (TSS) is not altered, the mutation would abolish the translational regulation of the 5'TOP element in the mutated allele. Therefore, we studied the effect of the recurrent mutation on the TSS of the gene. We performed rapid amplification of cDNA ends (RACE) by ligating a RNA linker to the 5' terminus of the mRNAs (Figure1B) to map the TSS. According to the results (Figure 1C), the recurrent mutation alters the TSS so that it initiates from a cytidine located at position Chr1:153963238. As a result, the 5'TOP element was not abolished but rather the pyrimidine stretch became longer, from 5 to 6 nucleotides (CTTTCC to CTTTTCC, respectively).

## DISCUSSION

Our comparative analysis of several next generation sequencing studies retrieved a novel recurrent mutation located at the TSS of the *RPS27* gene in ~10% of the melanoma cases. The *RPS27* gene is overexpressed in several malignancies, including melanoma [33, 34]. Moreover, *RPS27* is known to be involved in growth regulation and carcinogenesis [35, 36]. One of the signaling pathways that regulates 5'TOP bearing mRNA is the mTOR pathway, which is activated by growth factors and selectively inhibited by Rapamycin [30]. Previous studies have shown that mTOR is highly activated in the majority of melanoma cases and is associated with poorer prognosis characteristics of the disease [37,38]. In addition, it has been indicated that the length of the pyrimidine stretch of the 5'TOP element may affect its translational regulation efficiency [39, 40]. In light of these results and the RACE data presented in this study, we hypothesize that the mutation in the *RPS27* 5'UTR would promote the expression of the gene upon the induction of mTOR, which may contribute to the progression and/or the viability of the malignant cells.

In summary, this is the first study to demonstrate that melanoma patients harbor mutations in *RPS27*. At a mutation rate of ~10%, *RPS27* is ranked as the fourth most frequent gene with recurrent mutations identified in melanoma (ranked order: *TERT, BRAF*, *NRAS*, *RPS27*, *RAC1*). Functional studies of the effect of the recurrent mutation are ongoing. Yet, due to the high frequency of the mutation, we hypothesize that *RPS27* is a bona-fide driver of melanoma development.

## MATERIALS AND METHODS

### Data formatting

All mutational data from 6 different whole exome/genome sources was collated, 4 published studies [13, 18, 28, 29] as well as unpublished data. The data was first formatted to so that all positional data was mapped to the same genome build. In this case any data that was on hg18 was lifted over to hg19 using the Lift Genome Annotations tool available from UCSC (http://genome.ucsc.edu/cgi-bin/hgLiftOver). In some situations with the merging of data it became necessary to eliminate redundant information. For instance, with some samples both a tumor and a cell line derived from it were sequenced in which case the majority of the mutations were shared. This is also true in the case of samples shared between studies [28] as well as the multiple metastases extracted from the same patient in another study [18]. When removing these duplicates all mutations were retained at a count of one and the sample name was merged into a single entry. This step was taken to ensure that the number of recurrent positions was not inflated in later analysis. Once this list of mutations was established, the positional data and changes were formatted to an oncotator input format and annotated using the web-based version of oncotator (http://www.broadinstitute.org/oncotator). The next step taken was to remove any samples that were listed as acral, mucosal or uveal melanoma subtypes as the focus of this study was on cutaneous melanoma. In the final step we also removed any samples where the initial publication did not include a matched normal genotype. This left us with a list of 246 samples to investigate.

### Validation screen

Mutation detection was performed using the Sequenom MassARRAY® system following standard protocols and assays were designed using the Sequenom Assay Design suite online (Sequenom, San Diego). In brief, 20 ng of genomic DNA were used in a PCR reaction and cleaned post-amplification with shrimp alkaline phosphatase. A single base pair extension reaction using iPLEX Pro chemistry was performed, resin-treated to remove contaminants and spotted onto SpectroCHIP II arrays. Mutant and wild type alleles were then discriminated via mass spectrometry using the Sequenom MassARRAY® Analyser 4 platform. Mutations were detected using a minimum 10% threshold of the mutant allele peak and were all manually reviewed. The oligos used for the PCR, the extension stages and a BLAT analysis confirming that the amplified region is unique for *RPS27* gene are described in supplementary figure 2.

### Rapid Amplification of cDNA Ends (RACE) assay

Total RNA was extracted from 39T cells harboring the *RPS27*mutation using an RNeasy kit (Qiagen) according to the manufacturer instructions. RACE assay was performed using a GeneRacerTM Kit (Invitrogen). Briefly, 7 µg of total RNA were dephosphorylated using Shrimp Alkaline Phosphatase (New England, BioLabs). Then the sample was subjected to tobacco acid pyrophosphates (epicenter). An RNA linker with the sequence 5':CGACUGGAGCACGAGGACACUGACA UGGACUGAAGGAGUAGAAA was ligated to the 5' terminus of the mRNA using T4 RNA Ligase (New England, BioLabs). cDNA was prepared with a RSP27 gene specific oligo 5': AGTGCTGCTTCCTCCTGAAG using AMV reverse transcriptase, native (EURx). cDNA template was amplified with Platinume Taq DNA polymerase (Invitrogen) using the following primers: Forward 5' GGACACTGACATGGACTGAAGGAGTA,Reverse 5' CGTTTGTGCATGGCTAAAGA. The PCR products were cloned into the pGEM-T Easy vector. Colonies were picked and sequenced. Sequencing results were analyzed using Mutation Surveyor V3.10.

## SUPPLEMENTARY FIGURES, TABLES AND INFORMATION






