# Supplementary material for: A highly recurrent RPS27 5'UTR mutation in melanoma
Source: Oncotarget. 2014 Jun 1;5(10):2912–7. doi: 10.18632/oncotarget.2048 (PMC4102779; doi:10.18632/oncotarget.2048)
Supplement: Supplementary file 1 [file oncotarget-05-2912-s001.pdf]

Supplementary Figure 1

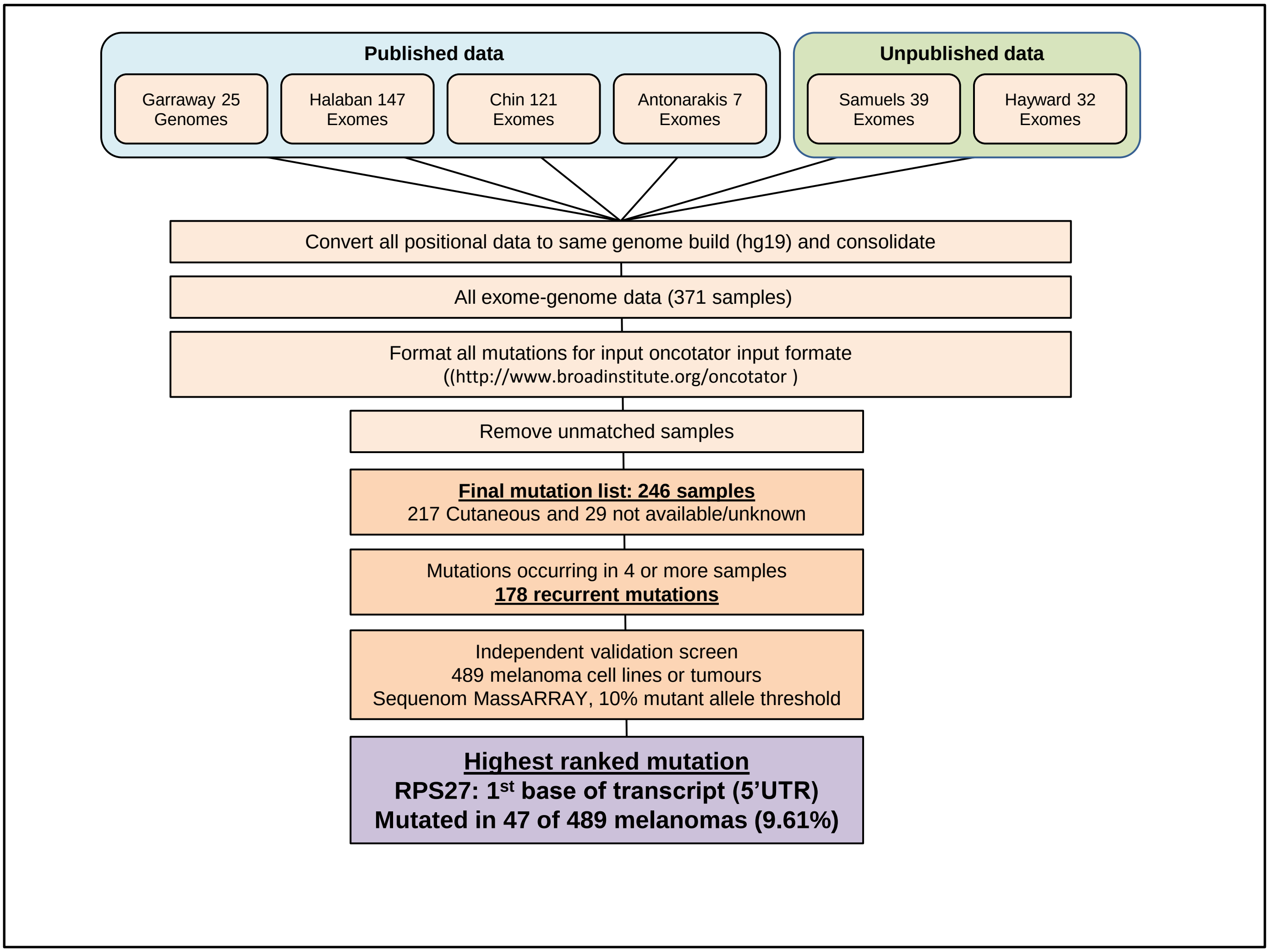

Supplementary Figure 2

A. Forward- 5’ ACGTTGGATGTCATTTCCTGTAGTGTGCTC

Reverse- 5’ ACGTTGGATGGCCGAAACCTGGACCAAAG

Extension- 5’ GAGGAGGTCGTCA CCGCCGGAAA

B.

chr1:153963178-153963314

chr1:153963239 ↓

```
GTCATTTCCTGTAGTGTGCTCtatataaggggcaggatttccgctttcgctcct  54
ttccggcggtgacgacctacgcacacgagaacatgcctgtgagtgCTTTGGTCC 108
AGGTTTCGGC                                              118
```

C.

SCORE	START	END	QSIZE	IDENTITY	CHR.	STRAND	START	END	SPAN
118	1	118	118	100.0%	1	+	153963187	153963304	118
38	53	92	118	97.5%	12	-	3321078	3321117	40
37	52	92	118	95.2%	11	-	116906762	116906802	41
35	54	92	118	94.9%	1	-	202441240	202441278	39
32	52	92	118	84.3%	1	+	38021815	38021853	39

Validation of the specificity of the oligos used for the sequenom analysis, to the mutation region of *RPS27* gene. **A.** description of the sequence used for sequenome analysis. **B.** the sequence of the amplified genomic region as obtained from the *in silico* PCR analysis. The sequence of the primers is described as Caps letters. The genomic location of the mutation is pointed by an arrow. **C.** BLAT analysis of the amplified region of *RPS27* gene used as a template for the extension step. The table describe the location and % of identity of the different homologous regions in the genome to the *RPS27* gene. The *in silico* PCR and the BLAT analysis were performed using the UCSC website (http://genome.ucsc.edu/index.html).
